# Emerging Insights Into the Role of Epigenetics and Gut Microbiome in the Pathogenesis of Graves’ Ophthalmopathy

**DOI:** 10.3389/fendo.2021.788535

**Published:** 2022-01-05

**Authors:** Yan Wang, Xiao-Min Ma, Xin Wang, Xin Sun, Ling-Jun Wang, Xin-Qi Li, Xiao-Yan Liu, Hong-Song Yu

**Affiliations:** ^1^ Department of Immunology, Special Key Laboratory of Ocular Diseases of Guizhou Province, Zunyi Medical University, Zunyi, China; ^2^ School of Basic Medical Sciences, Special Key Laboratory of Gene Detection and Therapy of Guizhou Province, Zunyi Medical University, Zunyi, China

**Keywords:** Graves’ ophthalmopathy, epigenetics, DNA methylation, histone modification, noncoding RNAs, gut microbiome

## Abstract

Graves’ Ophthalmopathy (GO) is an organ-specific autoimmune disease that is often characterized by infiltration of orbital tissues and is considered as the most common extra-thyroid manifestation of Graves’ disease (GD). Although genetic susceptibility has been found to be critical for the phenotype of GO, the associated risk alleles in a single gene are generally insufficient to cause the disease. Accruing evidence has shown that epigenetic disorders can act as the potentially missing link between genetic risk and clinically significant disease development. Abnormal epigenetic modifications can lead to pro-inflammatory cascades and activation of orbital fibroblasts (OFs) by promoting the various inflammatory response pathways and regulating the diverse signaling molecules that are involved in the fibrogenesis and adipogenesis, thereby leading to the significant expansion of orbital tissues, fibrosis and inflammation infiltration. Additionally, emerging evidence has shown that the gut microbiome can possibly drive the pathogenesis of GO by influencing the secretion of Thyrotropin receptor antibody (TRAb) and T-helper 17 (Th17)/regulatory T cells (Treg) imbalance. This paper describes the latest epigenetic research evidence and progress made in comprehending the mechanisms of GO development, such as DNA methylation, histone modification, non-coding RNAs, and the gut microbiome.

## Introduction

Graves’ ophthalmopathy (GO), also known as thyroid-related ophthalmopathy, is an autoimmune disease associated with Graves’ disease (GD) that involves orbital tissues. It has been found that about 25% of GD patients develop GO during their disease course (1, 2). Approximately 90% of GO develop in hyperthyroidism caused by GD, whereas GO sometimes occurs in patients with euthyroid or hypothyroid chronic autoimmune thyroiditis (3, 4). Although the ratio of female to male (F/M) has been found to vary based on the different studies, the incidence of GO is significantly higher in women than in men (5). The common clinical characteristics of GO are eyelid retraction, swelling, exophthalmos, retrobulbar pain, and even vision impairment resulting from optic neuropathy (6). Similar to GD, the autoreactive inflammatory process of orbital tissue plays a major role in the pathogenesis of GO. The extraocular muscles and connective tissue of GO patients are infiltrated by activated monocytes (such as T cells), as well as a small number of plasma cells, macrophages and mast cells (7). Activated T cells, mainly CD4^+^T cells, produce a large number of cytokines to amplify and maintain orbital inflammation and stimulate the proliferation of orbital fibroblasts (OFs) and the synthesis of glycosaminoglycans (GAG) (8). OFs are heterogeneous cells, and when activated, fibroblasts with a high level of thymocyte differentiation antigen 1(Thy1) surface expression (Thy1+) are more likely to form myofibroblasts, on the contrary, fibroblasts with little or no Thy1 (Thy1 -) are more likely to form adipocytes (9, 10). Accumulation of GAG leads to edema of extraocular muscles (11) and eventually leads to orbital swelling, tissue expansion and fibrosis development.

As mentioned above, cytokines and immune mechanisms play an important role in the pathogenesis of GO. CD4^+^T cells can be divided into auxiliary T helper 1(Th1), Th2, Th17, regulatory T (Treg) cells and other subtypes, which are considered to be related to the occurrence and development of GO (8). Studies have shown that Th1 cytokines are not only related to the progression of GO, but may play a dominant role in the orbital involvement in the early stage of GO. On the contrary, Th2 cytokines have no direct correlation with disease progression, but may play a greater role in the later stage of the disease (12–14). IL-17A secreted by Th17 cells has pro-inflammatory and pro-fibrosis effects, which further drives the clinical manifestation of GO (15). Treg cells are a subgroup of T cells with immune regulatory function, and impaired Th17/Treg balance may also be an important cause of GO (16). Furthermore, activated T cells activate the differentiation of B cells into plasma cells and the production of antibodies by secreting IL-4 (8). GD related hyperthyroidism is primarily caused by binding of TRAb to the thyrotropin receptor (TSHR) on the thyroid follicular endothelial cells, thereby stimulating the excessive secretion of the thyroid hormones. Furthermore, recent studies have reported that TRAb can effectively stimulate the proliferation of OFs, which is positively correlated with the clinical features of GO and can adversely affect the prognosis (17). Another crucial autoantigen involved in the pathogenesis of GO is insulin-like growth factor I receptor (IGF-IR), which forms a physical and functional complex with TSHR in OFs from GO patients (18). Antibodies of the IgG class generated by GD patients are able to bind to IGF-1R and initiate the signaling from the TSHR/IGF-IR protein complex. Furthermore, Teprotumumab, an IGF-IR blocking monoclonal antibody, has been approved in the US FDA for the treatment of GO and works by attenuating signaling from TSHR or IGF-IR (19). Currently, substantial progress has been made in treating GO, but the moderate-to-severe GO patients usually do not completely respond to available medical treatments, and complete *restitutio ad integrum* almost never occurs (5, 20). Therefore, more extensive studies are needed to explore the pathogenesis of GO and develop prompt prediction, timely referral and novel therapeutic strategies for GO.

In the past decade, increasing evidence has shown that genetic susceptibility plays a crucial role in the pathogenesis of GD and GO (21–24), but the associated risk alleles in a single gene are generally insufficient to cause disease. Moreover, several studies have performed Human leukocyte antigen *(HLA)*, cytotoxic T lymphocyte antigen-4 *(CTLA4)*, Interleukin (IL) - 23 receptor *(IL23R)*, and *TSHR* genotyping on GO and non-GO patients in GD patients. The results show no statistical differences in allele and genotype frequency between the two groups, thereby plausibly suggesting that GO might not have significant genetic susceptibility in GD patients (25). These results suggested that epigenetics and/or environmental influences could possibly also play an important role in the pathogenesis of GO. Epigenetics as a research hotspot in recent years, researchers have found that the decreased level of *ICAM1* methylation is significantly associated with exophthalmos in patients with GD (26). Similarly, micro RNAs (miRNAs) have been correlated with the level of autoimmune antibodies such as TPOAb, TgAb and TRAb (27). These findings provided us with vital background to explore the potential pathogenesis of GO. A number of previous epigenetics related studies have shown that different environmental factors can also functionally regulate gene expression and phenotypes in the disease development process without altering the DNA sequences, primarily through epigenetic mechanisms such as those of DNA methylation, histone modification, and non-coding RNAs (28). Epigenetic modifications have been found to be involved in the dysregulation of the different signaling molecules and receptors in a variety of autoimmune/inflammatory conditions, including GO (29–32). Thus, epigenetic studies could serve as interesting targets for elucidating the pathogenesis of GO. Interestingly, all environmental factors involved in the pathogenesis of GD and GO can effectively alter the balance of microorganisms located in the gut and influence the immune system, particularly the proportion of Treg and Th17 cells. A recent study demonstrated that gut dysbiosis can lead to the imbalance of Th17/Treg through the propionic acid regulated pathway, and promote the occurrence of GD (33). A number of studies in GD/GO mice model have also shown that the gut microbiome might induce the clinical manifestation of GO by influencing the levels of TRAb and Th17/Treg imbalance (34, 35). Therefore, this paper reviews the latest epigenetics landscape and progress made in deciphering the mechanisms of GO by encompassing the various aspects of DNA methylation, histone modification, noncoding RNAs and gut microbiome, with an aim to provide new effective potential therapeutic strategies for the management of GO.

## Abnormal DNA methylation and GO

As one of the most common forms of epigenetic modification, DNA methylation is catalyzed by DNA methyltransferases (DNMTs) by using S-adenosyl-L-methionine (SAM) as a methyl donor, and forming 5-methylcytosine (5-MC) by the transfer of the methyl group to the five carbon atoms of the CpG dinucleotide cytosine base. Accumulating evidence has suggested that DNA methylation in the promoter regions of the CpG island is primarily linked to gene transcriptional inhibition; and the methylated CpG binding domain (MBD) family can effectively recognize and bind to methylated CpG to cause significant transcriptional inhibition (36).

**Figure 1:**
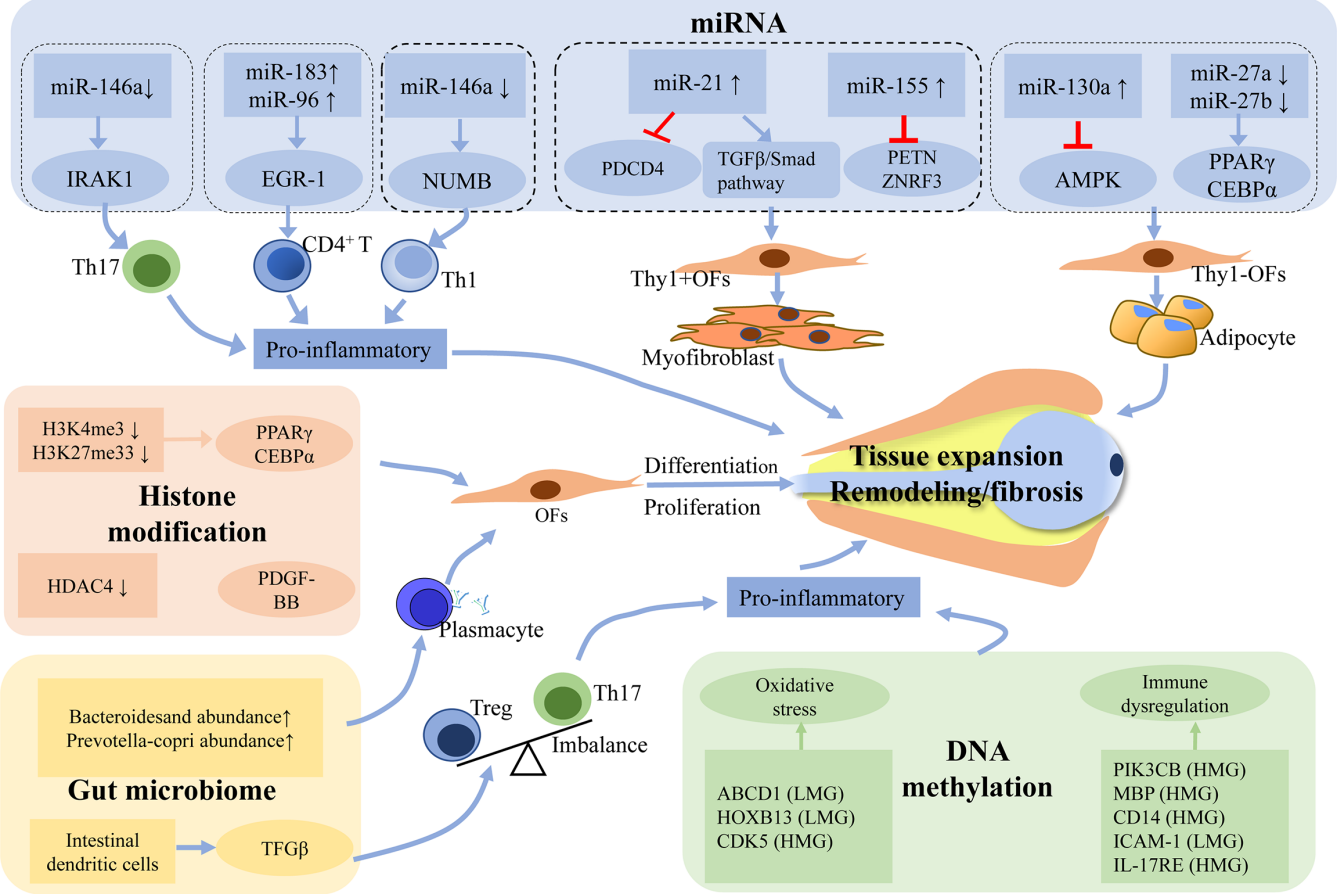
Potential mechanism of epigenetics and gut microbiome in the pathogenesis of GO. Abnormal epigenetic modifications may promote pro-inflammatory cascades and disrupt the expression of signaling molecules that are involved in the fibrogenesis and adipogenesis of orbital fibroblasts. Gut microbiome possibly drives the pathogenesis of GO by influencing secretion of TRAb and Th17/Treg imbalance. The process of all these changes will lead to the hyaluronan production and the activation of orbital fibroblasts, which eventually develop into tissue expansion, fibrosis and inflammation. LMG, lowly methylated genes; HMG, highly methylated genes; TRAb, Thyrotropin receptor antibody; Treg, regulatory T; Th17, T-helper 17.

In recent years, several studies have shown that DNA methylation exhibits important functions in the pathogenesis of GO by altering the gene expression profile. Xin et al. (37) have identified 841 differentially methylated sites by sequencing the whole wide genomic DNA methylation in 6 patients with GO. It was found that 148 different genes were located near or at the methylation sites. These genes include various genes mediating immune responses such as Cluster of Differentiation 14 *(CD14)*, interleukin 17 receptor E *(IL17RE)*, beclin1*(BECN1)*, cyclin dependent kinase 5 *(CDK*5), oxidative stress regulation genes including ATP-Binding cassette transporter superfamily D1 *(ABCD1)*, homeobox B13 *(HOXB13)*, thyroid function regulating gene such as dopamine receptor D4 *(DRD4)*. The aforementioned genes *IL17RE* and *CDK5* have been positively associated with Clinical Activity Score (CAS) and TRAb, respectively. The authors also performed enrichment of genes associated with the diverse biological processes, cellular composition, and molecular functions. To further understand the pathogenesis of GO, pathway analysis of these identified genes was carried out, and four potentially important pathways, including toxoplasmosis pathway, axon guidance pathway, focal adhesion pathway, and proteoglycans in cancer pathway, were found to be closely associated to the occurrence and development of GO. Moreover, other important pathway genes such as *LDLR*, *CDK5*, and phosphatidylinositol-4,5-bisphosphate 3-kinase catalytic subunit beta (*PIK3CB)* that play an important role in regulating inflammatory have also been found to be significantly correlated with GO phenotypes (32). These observations further confirmed the potential relationship between the DNA methylation level and its functions in the regulation of specific genes involved in the pathogenesis of GO. In another study, Shi et al. (38) carried out genome scale screening of DNA methylation in the peripheral blood samples of patients with GO and healthy controls. They next screened the different candidate genes with a good topological performance by constructing a gene regulatory network of hypermethylated and hypomethylated genes. They further expanded the sample for verification by monomethylated sequencing, thus confirming that, the methylation level of boule homolog *(BOLL)* was lower, and the methylation level of myelin basic protein *(MBP)* and *CDK5* was markedly higher. *BOLL*, *MBP*, and *CDK5* genes were found to be associated with increased risk of GO. The possible mechanism can be described by the fact that *MBP* is related to the activation of *MBP* specific CD8 lymphocytes and *CDK5* could promote oxidative stress. Khong et al. (39) used microarray analysis to detect the gene expression profile of orbital adipose tissue obtained from GO patients. In combination with gene set enrichment analysis (GSEA), they found that compared with inactive GO, the gene enrichment of active GO showed significantly more prominent epigenetic characteristics of acute myeloid leukemia (AML). Moreover, the abnormal hypomethylation of epigenetic markers in AML was associated with immunodeficiency signaling, cytotoxic T cell-mediated apoptosis, and dysregulation of T cell receptor signaling (40). In summary, the pathogenesis of GO is driven by aberrant DNA methylation of specific genes such as ABCD1 and PIK3CB, which may cause oxidative stress and immune dysregulation leading to inflammation (**Figure 1**).

Furthermore, OFs play a crucial role in the initiation and maintenance of the inflammatory response as well as in orbital tissue expansion and remodeling. Hu et al. (41) performed DNA methylation sequencing and orbital computer tomography (CT) measurement on the peripheral blood samples from patients with GO and healthy controls. They found that the abnormal methylation of *MBP* gene was significantly correlated with the ratio of the total cross-sectional area of the orbital muscles (OM) to the total orbital area in GO patients. Virakul et al. (42) similarly carried out the detection of various OFs, and further reported that OFs in active versus inactive GO patients might have different proteomic and DNA methylation characteristics, thereby indicating that OFs can show specific pathological characteristics that might be related to the disease stage during the progression of GO. These observations highlighted the potential role of OFs as a future therapeutic target in GO. These findings also suggested that DNA methylation occurs in the fibroblasts and epigenetic understanding of the transition of OFs from “inflammatory/pre-lipogenic” to “remodeling/pre-fibrotic” effector cells should be a critical area of research in the future studies.

Genetic polymorphisms of a few selected DNA methylation regulatory genes can also lead to abnormal DNA methylation and dysfunction of these genes, which can further increase the susceptibility of the host (43). For example, methylenetetra-hydrofolate reductase *(MTHFR)* reduces 5, 10-methylenetra-hydrofolate to 5-methylenetrahydrofol -ate in the folic acid metabolic pathway, thus extending to the methyl transport pathway and indirectly facilitating methyl groups for the DNA methylation and protein methylation through the re-methylation of homocysteine residues. Lee et al. (44) reported that *MTHFR* polymorphism was closely associated with GO susceptibility and that *MTHFR 677 TT* can serve as an independent risk factor for GO. Moreover, SNPs *677C>T* of the *MTHFR* gene have been demonstrated to result in pronounced alteration of its enzymatic activity (45), providing indirect evidence for the important role of DNA methylation in GO pathogenesis.

Taken together, DNA methylation plays an important role in the occurrence and development of GO, but the related research is rather limited. Therefore, more studies are needed to explore the key role of DNA methylation in the pathogenesis of GO, to develop biomarkers for the rapid detection of early DNA methylation changes and establish effective GO treatment strategies.

## Histone Modification and GO

The nucleosome constitutes the basic structural unit of chromosomes. It consists of an octamer composed of four different pairs of core histones (H3, H4, H2A, and H2B) and a 147bp length of the double-stranded DNA surrounding the octamer. The core histones moieties are generally spherical, and the non-structural N-terminal amino acids extended by each histone can be modified after translation. The common histone modifications events include methylation, acetylation, phosphorylation, ubiquitination, adenosine diphosphate (ADP) ribosylation, etc. (46). Currently, histone modification studies have primarily focused on the methylation and acetylation, thereby providing some evidence for the possible role of histone modification in regulating immune tolerance and autoimmune thyroid diseases (47–49).

Histone methylation can be catalyzed by histone methyltransferase (HMT) and occurs mainly on the lysine and arginine residues of H3 and H4 histones, but the transcriptional output from histone methylation primarily depends on the nature of the specific residues involved. Histone H3 methylation is one of the most well characterized epigenetic modifications and actively involved in the adipogenesis of OFs. The differentiation of OFs into adipocytes is primarily a peroxisome proliferator-activated receptor gamma (*PPARγ*) and CCAAT/enhancer binding protein alpha (*CEBPα*) dependent process (50). Khong et al. (39) conducted GSEA on the expression profile of the various differentiated genes in the active phase of GO and found that the unmethylated histone H3 in the genes with high CpG density promoters (HCP) found in embryonic fibroblast was significantly downregulated compared with the normal controls. Methylation at K4 (H3K4me3) and K27 (H3K27me33) on histone 3, which is in bivalent status on HCP has been directly related to cell lineage commitment. *PPARγ* often remains in a bivalent state during embryonic fibroblast differentiation (51). Matsumura et al. (52) reported that the H3K4me3 and H3K27me3 bivalent state might change to H3K4/H3K9me3 during lineage specification. The characteristics of H3K4/H3K9me3 bivalent chromatin signature can possibly maintain *CEBPα* and *PPARγ* gene expression at a very low level in lineage-committed mesenchymal stem cells (MSCs) and preadipocytes by silencing or by maintaining developmental genes to poise for subsequent activation, and they may constitute a distinct pseudo heterochromatin/euchromatin boundary which is important for adipocyte differentiation. The above studies suggested that histone H3 methylation might play an important role in the pathogenesis of GO by targeting some genes that are related to adipogenesis. However, at present, there are only few related studies, and there are no reports to establish that abnormal histone methylation might be related to the occurrence of GO. Therefore, additional studies are needed to decipher the specific mechanisms underlying these observations.

Histone acetylation and deacetylation are important components of the gene regulatory machinery. Histone acetylation is usually catalyzed by histone acetyltransferase (HAT), and addition of acetyl groups to the target histones can activate the various transcription factors which can specifically bind to the different DNA binding sites and promote gene transcription. Histone deacetylase (HDAC) can also catalyze the deacetylation of histones to make DNA more tightly encapsulated around the nucleosomes, thus inhibiting gene expression (46). Histone acetylation and deacetylation can effectively maintain a dynamic balance in the nucleus. For example, loss of HDAC expression in the thyroid and immune cells of patients with autoimmune thyroid diseases has been confirmed (53). Recently, Ekronarongchai et al. (31) reported HDAC4 mRNA upregulation and protein expression in OFs by Platelet-Derived Growth Factor-BB (PDGF-BB). It was observed that the expression of HDAC4 mRNA in the OFs of GO patients was significantly higher as compared to the healthy control group, and the lysine 9 acetylation of histone H3 (H3K9ac) was markedly decreased under PDGF-BB stimulation. It was also found that the expression of hyaluronan synthase 2 (*HAS2)*, collagen type I alpha 1 chain (*COL1A1)*, proliferation marker Ki67 *(Ki67)*, and α-smooth muscle actin *(α-SMA)* mRNA and the production of hyaluronic acid in PDGF-BB stimulated OFs were significantly decreased upon HDAC4 silencing. These data suggested that HDAC4-induced H3K9 deacetylation could potentially exacerbate GO OFs hyperproliferation and extracellular matrix production by functionally regulating PDGF-BB, thus suggesting that HDAC4 might serve as a new target for GO therapy.

Therefore, histone modification has been related to the occurrence and development of GO. Histone H3 methylation and HDAC may be potential targets for GO therapy, but the specific mechanisms are still not clear. More studies are needed to identify abnormal histone modifications associated with GO and to clarify their molecular mechanisms in GO.

## Non-Coding RNAs and GO

Non-coding RNAs are a class of RNA transcripts that lack the function of coding proteins. They can be classified into miRNAs, circular RNAs (circRNAs) and long noncoding RNAs (lncRNAs), which have been reported to play an important role in the pathogenesis of the various autoimmune thyroid diseases (54).

### MiRNAs and GO

MiRNAs are endogenous small non-coding RNA molecules that contain 18 to 23 nucleotides in length, and that by binding to the untranslated 3’ domain of the target gene mRNA, miRNAs can effectively mediate the degradation of mRNA or inhibit its translation, thus exerting a strong negative regulatory effect on the gene expression at the post-transcriptional level (55). MiRNAs can regulate more than 60% of protein- coding genes in the humans and have been implicated in the various biological processes such as the lineage commitment in the immune system, cell proliferation, differentiation, apoptosis, and maintenance of immune homeostasis (56, 57). An abnormal expression of miRNAs has been closely related to the pathogenesis of various autoimmune-mediated eye diseases such as GO (58). Circulating miRNAs can be detected in the peripheral blood and orbital tissues of GO patients, with different expression levels in the active disease stage of GO.

#### Circulating miRNAs and GO

A number of recent studies have shown that some circulating miRNAs might be directly involved in the development of GO, and have been found to be closely related to the regulation of differentiation or activation of immune cells and immune responses.

MiR-146a-5p is one of the most widely studied miRNAs and has an important role in regulating GO progression. MiR-146a is a typical multifunctional miRNA whose expression can be induced by toll-like receptor 4 (TLR4)/nuclear factor-kappa B (NF-ĸB) pathway, which acts as an important regulator of immune and inflammatory response pathways (59). By targeting tumor necrosis factor receptor-associated factor 6 (TRAF6) and IL-1 receptor-associated kinase 1 (IRAK1), miR-146a can negatively inhibit the feedback of NF-ĸB signaling cascade and modulate the signal transduction by the related receptors and subsequent cytokine release (60, 61). A few studies have shown that the peripheral blood level of miR-146a-5p in GO patients was significantly decreased as compared with their healthy counterparts as well as GD patients which lack GO phenotype (62–64). Additionally, reduced expression of miR-146a-5p has been negatively correlated with the CAS and IL-17 levels in GO patients. IL-17 levels have been positively correlated with CAS, and the downregulation of miR-146a can effectively lead to a paradoxical increase in IL-17 levels possibly due to their inability to inhibit IRAK1. This finding suggested that IRAK1 could play a key regulatory role in the differentiation of Th17 cell (62). The orbital tissues are infiltrated by activated mononuclear cell such as CD4^+^ T cells, which exert a key role on GO activity (7). A number of recent studies have also suggested that the down-regulated expression of miR-146a in CD4^+^T cells of GO patients might facilitate GO development by targeting NUMB genes, thus resulting in the development of inflammatory Th1 response, which can lead to orbital inflammation in GO patients (63, 64). In other words, the downregulation of circulating miR-146a may indirectly promote the development of GO by regulating the expression of various inflammatory mediators. Contrary to the downregulation of miR-146a, miR-155 levels have been reported to be increased in the peripheral venous blood in GO patients. In turn, both miR-155 and miR-146a have multiple target genes in the TLR4/NF-ĸB pathway, which can mutually regulate the immune response. The upregulation of miR-155 may also promote autoimmune inflammation through the targeted inhibition of cytokine signaling 1 (SOCS1) and SH2 domain-containing inositol-5-phosphatase 1 (SHIP1) (65). Other miRNAs such as miR-Let7d-5p, miR-96-5p and miR-301a-3p, were also found to be differentially expressed in the serum of GO patients versus control group, which might contribute to the regulated expression of the different inflammatory mediators *in vivo* and can lead to the occurrence as well as the development of GO (27). In addition, Thiel and colleagues (66) have reported an increased expression of miR-183 and miR-96 in CD4^+^T cells of GO patients and in activated T cells of mice. It has been found that these two kinds of miRNAs promote the activation of CD4^+^T cells by effectively regulating the expression of early growth response 1(EGR-1), which can directly be related to the activation of PTEN/Akt signal axis, and thus participate in the occurrence as well as the development of GO. At present, based on the special characteristic of the differential expression of miRNAs, it will be of great significance to study the role of the various influencing factors involved in the pathogenesis and to decipher the mechanisms of the transition from an active stage to static stage, to facilitate early diagnosis and treatment possibilities of GO.

The clinical utility of miRNAs in GO has also been explored. For instance, Shen et al. (67) found that the low serum miR-224-5p could be correlated with GO glucocorticoid (GC) insensitivity, and overexpression of miR-224-5p *in vitro* increased GC sensitivity and glucocorticoid receptor protein *via* targeting glycogen synthase kinase 3 beta. Moreover, the combination of baseline serum miR-224-5p and TRAb was an independent risk factor for GC insensitivity. Recently, a number of studies have analyzed the possible role of miRNAs in the assessment of GO severity and disease outcomes. One study by Martinez-Hernandez et al. (27) reported that miR-let7D-5P expression was markedly decreased in GO patients as compared with GD patients without GO and was also found to be negatively correlated with CAS. Meanwhile, Zhang et al. (68) performed miRNAs profiling of the thyroid tissues in GO patients using next-generation sequencing (NGS) and then identified their serum expression. They found that novel:19_15038 and hsa-miR-27a-3p were up-regulated in GO, while hsa-miR-22-3p was down-regulated in GO as compared to healthy controls. To sum up, the detection of the various differentially expressed miRNAs might be used as the potential biomarkers to predict the development and progression of GD into GO.

#### Orbital Tissue miRNAs and GO

OFs are both the target cell and effector cell in the pathogenesis process of GO. A number of recent studies have suggested that miRNA inhibitors or mimics can be used to up-regulate or down-regulate miRNAs expression in orbital tissues, thus regulating the corresponding biological behavior. Recent studies have also indicated that some miRNAs might be associated with orbital fibrosis, adipose tissue formation and inflammatory cell development.

For example, miR-146a acts as a key regulatory factor of GO orbital tissue fibrosis. To our knowledge, the level of miR-146a might be variable in the different types of tissues (63, 69). Unlike the downregulation of miR-146a in peripheral blood summarized previously, it is mostly up-regulated in orbital tissues. Wang et al. (69) reported that up-regulation of miR-146a in the orbital connective tissue of GO patients could significantly increase IL-6 level by inhibiting Notch2, thereby effectively promoting mitotic activity of fibroblasts in GO patients, inhibiting cellular apoptosis, and subsequently worsening the deterioration of GO. Woeller et al. (70) reported that TSHR signaling could induce the production of miR-146a and miR-155 on the OFs of GO patients. The expression of the different cell proliferation suppressor genes *ZNRF3* and *PTEN* were substantially decreased, thereby indirectly promoting the proliferation response of OFs, which could possibly explain the pathological mechanisms of partial fibroplasia observed in GO. However, a few studies have also shown that miR-146a can inhibit the fibrosis process. MiR-146a also plays an active role in the anti-inflammatory and anti-fibrotic processes of OFs. Jang et al. (71, 72) have reported that inflammatory stress can significantly up-regulate miR-146a expression in OFs, but inhibit the expression of IL-6, ICAM-1, and other inflammatory proteins. It can also negatively regulate levels of transforming growth factor beta (TGF-β) -induced fibrosis markers such as fibronectin (FN), collagen I α, and α - SMA. Type I collagen is considered to be a critical marker of fibrosis, and hyaluronic acid is a glycosaminoglycan. Liu et al. (73) recently reported that miR-146a expression was markedly down-regulated in the secretion of hyaluronic acid and type I collagen in GO OFs *in vitro*, which can reduce the aggregation of glycosaminoglycan and collagen deposition, and thereby delay the progression of the disease. Li et al. (65) hypothesized that miR-155, in contrast to miR-146a, can enhance the development of inflammatory T cells by promoting different autoimmune responses. In particular, the levels of miR146a in circulating and orbital tissues have been found to be inconsistent, which may be because the inflammation caused by miR-146 in peripheral blood may not fully reflect the inflammation in orbit. What is more, the contradictory functions about the relationship between miR-146a and differentiation of OFs can also be difficult to interpret, mainly due to the manifestation of the different targeting effects. Furthermore, miR-146a was found to play different functions in the regulation of the diverse signaling pathways or in interactions with the different target mRNAs. However, elucidation of the detailed mechanism of miR-146 in GO requires further investigation.

In addition to miR-146a, miR-21 has also been reported to play an important role in orbital muscle fibrogenesis. Lee et al. (74) found that in human OFs, PDGF-BB can significantly inhibit the expression of Programmed cell death factor 4 (PDCD4) by up-regulating miR-21, and thus promoting the proliferation of OFs. It is consistent with the study that the translation of PDCD4 was inhibited by miR-21 by Young et al. (75). Therefore, PDGF-BB/miRNA-21/PDCD4 pathway might form the basis of novel strategies for developing therapeutic interventions against GO. Tong et al. (76) reported that expression of miR-21-5p in GO eye fibroblasts was markedly higher than that of the control group and that it could enhance TGF-β 1-induced expression of total type I collagen and mRNA level of type I collagen. In addition, anti-miR-21 not only blocked decapentaplegic3 (Smad3) phosphorylation but can also mimic activated Smad3 phosphorylation. These results suggested that miR-21 could substantially enhance Smad3 phosphorylation and activate TGF-β/Smad signaling pathway to participate in orbital muscle fibrogenesis. In summary, these studies indicated that miR-21 may provide new targets for the treatment of GO.

Adipogenesis has been found to be closely related to the degree of ocular protruding (77). Therefore, efforts are being made to find effective ways to block the adipogenesis pathway as a new therapeutic option. Hammond et al. (78) reported that when Thy1- OFs were accumulated in GO, the miR-130a level was significantly increased, resulting in the decreased AMPK activity and promotion of the fat accumulation in orbit. Moreover, the expression level of miR-27a and miR-27b in the orbital adipose tissue of GO patients was significantly lower than that of the controls, which explains the inability to negatively regulate the mRNA expression of the various adipogenic genes such as *PPARγ* and *CEBPβ* in GO fibroblasts, thus indirectly causing the adipogenic differentiation of OFs (79). Therefore, blocking orbital muscle fibrogenesis and adipose generating pathways through modulating miRNA expression could provide novel therapeutic options.

In summary, multiple miRNAs can play an important role in the occurrence as well as the development of GO, and especially miR-146a may serve as an important target (**Table 1**). However, the therapeutic applications of miRNA have not been fully developed, and hence more studies are needed to clarify the possible effects of miRNAs on other target genes in GO or its pathogenesis. In the future, miRNAs are expected to aid significantly in the precise diagnosis and targeted therapy of GO.

**Table 1:** Non-coding RNAs in the pathogenesis of GO.

Noncoding-RNAs	Samples/cells	Expression change	Function	Effects in GO	References
miR-146a	Plasma	Downregulation	Promote the differentiation of Th17 cell	Pro-inflammatory	(62)
	CD4+ T	Downregulation	Increase Th1 response	Pro-inflammatory	(63, 64)
	Orbital tissue	Upregulation	Increase the IL-6 level	Exacerbate GO	(69)
	Orbital tissue	Upregulation	Decreases FN, collagen Iα, and α -SMA	Inhibit fibrosis process	(72, 73)
miR-183 and miR-96	CD4+ T	Upregulation	Contribute to the activation of CD4+ T cells	Pro-inflammatory	(66)
miR-21-5P	Orbital tissue	Upregulation	Promote collagen Iα and total collagen production	Promote fibrogenesis	(76)
miR-146a and miR-155	Orbital tissue	Upregulation	Reducing the expression of PTEN and ZNRF3	Promote proliferation	(70)
m iR-27a and miR-27b	Orbital tissue	Downregulation	Cause the adipogenic differentiation of OFs	Promote adipogenesis	(79)
m iR-130a	Orbital tissue	Upregulation	Enhance lipid accumulation	Fatty tissue accumulation	(78)
circRNA_14940	Orbital tissue	Upregulation	Regulate the Wnt signaling pathway, ECM receptor interaction and PIK3-AKT signaling pathway	Participate in the pathogenesis of GO	(80)

### Other Noncoding RNAs and GO

CircRNAs are endogenous entities formed by the covalent binding of 3’ and 5’ end reverse splicing, which can display important biological functions in acting as miRNA sponge adsorption, attaching to the different RNA-binding proteins, and participating in protein translation (81, 82). However, the role of circRNAs in GO development is still in its infancy. For example, Wu et al. identified (80) 163 differentially expressed circRNAs from the orbital fat/connective tissues of GO patients. Through circRNA-mRNA co-expression and circRNA-mRNA interaction analysis, possible crosstalk of circRNA_14940 with down-regulated mRNA tenascin XB *(TNXB)* and up-regulated mRNA cyclin D1(*CCND1)* was analyzed and it was observed that abnormal regulation of the Wnt signaling pathway, PI3K-Akt signaling cascade, and extracellular matrix (ECM) receptor might also be involved in the pathogenesis of GO. CircRNA_10135 can also interact with prostaglandin F receptor (PTGFR) through modulating the calcium signaling pathway and participating in the adipogenesis process of GO. Meanwhile, circRNA_14936 has been found to be correlated with up-regulated mRNA TNF receptor superfamily member 19 (TNFRSF19), and hsa-miR-10392-3p that may potentially affect the occurrence and development of GO by regulating the interactions between circRNA_14936 and TNFRSF19 which can influence B cell survival. Therefore, circRNAs might play an important role in the pathogenesis and progression of GO (**Table 1**). However, there are only few studies conducted to evaluate the role of circRNAs in the pathogenicity of GO, so the functions of most circRNAs are not clear and their molecular mechanism remains to be further elucidated.

LncRNAs are an important class of non-coding RNAs with a length of more than 200 nucleotides. LncRNAs can regulate the gene expression through affecting various mechanisms, including those related to epigenetics, transcriptional, post-transcriptional, and miRNAs regulation. A number of previous studies have reported that lncRNAs may play an important role in the pathogenesis of GD (83). For instance, Christensen et al. (84) reported the first lncRNA Heg to be associated with GD and showed that was negatively correlated with monocyte CD14 mRNA and serum TRAb level in GD patients. Moreover, anti-thyroid therapy could not change lncRNA Heg level in GD patients. The intimate temporal relationship between the onset of GD and GO also clearly suggests that these two diseases may have the same underlying etiology. Xia et al. (85) identified a novel lncRNA named Lnc-Smad3, which can maintain the tight chromatin structure of Smad3 promoter through direct interaction with HDAC1, thereby inhibiting Smad3 transcription and affecting inducible Treg (iTreg) cell polarization through modulating the TGFβ/Smad3 signaling pathway.

LncRNAs are also involved in the development of Treg cells, which provides a new direction to further study of the possible relationship between Lnc-Smad3 and GO. Although there are some difficulties in screening and studying lncRNAs, identifying their role and mechanism in GO may be a necessary direction for future research.

## Gut Microbiome and GO

The human gut microbiome consists of about 100 trillion different gut microbial cells and serves as the main entry point for bacteria. In a symbiotic manner, the human host primarily provides the gut microbiota with crucial nutrients for promoting growth. In contrast, the gut microbes play an important role in the food digestion, detoxification, protection from pathogens, and the regulation of the host immune system. In fact, the highest concentration of T cells in the human body is located in the intestinal mucosa, where lymphocytes are primed to respond to the various microorganisms. Furthermore, the intestinal microbiome can also effectively regulate the local intestinal immune system and the systemic immune response (86, 87).

Moreover, multiple human and animal studies have shown that gut microbiome have been closely related to a variety of autoimmune diseases, such as inflammatory colitis (IBD) (88), rheumatoid arthritis (RA) (89), type I diabetes (T1D) (90), and systemic lupus erythematosus (SLE) (91). For instance, Jiang et al. (92) sequenced the fecal samples of patients with GD and healthy controls, and found that the richness of intestinal microflora in patients with GD decreased significantly. This alteration was characterized by a significant increase in *Bacteroides*, which damaged intestinal barrier function, resulting in the release of a large number of pro-inflammatory factors outside the intestine, thus resulting in immune dysfunction. In addition, it was also found that changes in thyroid hormone levels can lead to an increase in the number of *Lactobacillus.* It may play a harmful role in GD by activating NF- κB signaling pathway and autoimmune response to thyroid gland. At present, there are only few studies on intestinal flora in GO. Several reports have suggested that there is a marked decrease in bacterial community diversity in GO. Shi et al. (93) sequenced the fecal samples of patients with GO and healthy controls and found that compared with the control group, the proportion of *Bacteroidetes* and *Prevotellaceae* in patients with GO at different levels increased significantly, while the proportion of *Blautia* and *Fusicatenibacter* decreased significantly. The proportion of the *Succinivibrionaceae* and *Subdoligranulum* was positively correlated with TRAb, and there was a negative correlation between *Parabacteroides_disasonis* and TRAb. Further analysis showed that *Prevotellaceae* could clearly distinguish GO patients from the control group. The level of *s_Prevotea_copri* was reported to be positively correlated with the level of serum TRAb, which may be related to active orbital inflammation (94). Thereafter, the researchers conducted a comparative analysis of GD, GO and healthy people for the first time. They found that the proportion of *Deinococcus-Thermus* and *Chloroflexi* in patients with GO was significantly lower than that in GD (95). Interestingly, after transplanting the normal human feces into type 2 diabetic mice, researchers found that the abundance of *Bacteroides* and some bacteria decreased and blood glucose levels recovered. This led to the speculation that it may be possibly increasing the diversity of intestinal microflora that can affect the different types of microorganisms in the intestines of mice to achieve the effect of treatment (96). Therefore, whether fecal microbiome transplantation can also have a therapeutic effect on GO can be an important direction for future research. Additional in-depth studies about the role of intestinal microorganisms in GO will possibly provide new methods and ideas for the treatment of GO. In addition, some studies have reported that Salmonella that can cause enteritis possesses an antigenic cluster similar to the thyroid stimulating hormone receptor protein, where antibodies against the thyrotropin receptor proteins might be produced after infection (96). These results laid a sound foundation for exploring the interaction between intestinal microorganisms and TRAb. However, the current findings may also be related to thyroid autoimmunity rather than GO, and might not be entirely specific for GO.

Masetti et al. (34) used intramuscular injection of eukaryotic expression plasmid (thyroid stimulating receptor) in two distinct experimental centers in two different countries (Center 1 and 2). The GD/GO disease model of BALB/C female mice was established by immunological method of TSHR. The results showed that only BALB/C mice from Center 2 displayed hyperthyroidism and apparent changes in orbital tissues. Interestingly, it was found that the abundance and species composition of intestinal flora were significantly different between BALB/C mice tested from the two centers by 16S rRNA gene sequencing and traditional microbial culture. In Center 2, the intestinal flora structure of different intervention groups (TSHR group, β Gal group) was analyzed by 16S rRNA gene sequencing and it was found that the intestinal flora of TSHR group changed significantly (increased relative abundance of *Lactobacillus*, decreased relative abundance of *bacteroides*, etc.). These alterations were also associated with some clinical features such as thyroxine levels (fT4), TRAb, orbital adipogenesis, and muscular atrophy. The same group constructed another GD/GO model by using BALB/c and C57BL/6J mice. The results indicated that the C57BL/6 mice had higher TRAb, but compared with BALB/c mice, the spleen T cells of C57BL/6 mice exhibited poor proliferation of TSHR response, and secreted mainly anti-inflammatory cytokines such as IL-10, without elevated thyroxine levels or any orbital lesions, while resembling immunized BALB/C mice showed orbital pathology. These observations suggested that there was a different pattern of correlation between the microbiomes and disease (endocrine and immune) characteristics between the two different mouse strains (97). In addition, Shi et al. (94) found that operational taxonomic units (OTUs) derived from the *bacteroidesstercoris* species were markedly associated with the thyroglobulin autoantibodies, whereas OTUs from the *bacteroides* genus were associated with CAS. Masetti et al. (98) deciphered that the effect of intestinal microorganisms on the immune system can be primarily revealed by maintaining a balance between the Tregs and pro-inflammatory Th17 cells and Th1 cells. When the microbiome might not be ideal, intestinal dendritic cells can secrete TGF-β, which can cause the Th17/Treg imbalance, thus leading to the loss of immune tolerance. In individuals with a particular genetic predisposition, the result can potentially lead to GD and GO. These results clearly suggested that the gut microbiome could play a critical role in shaping the immune response that might significantly influence the TRAb levels and immune tolerance, thereby influencing thyroid and eye disease phenotypes.

The above studies have provided a novel perspective research pathway to clarify the potential mechanisms of intestinal microbiome disorders observed in GO. Hence, understanding that how the composition of the gut bacterial microbiome and the abundance of specific bacterial strains might affect the autoimmune response in GD and GO remains the next important aspect.

## Conclusion

With the emergence of novel bioinformatics tools and high-throughput gene sequencing technologies, the important role of epigenetics and gut microbiomes in GO has been gradually elucidated (**Figure 1**). Still, several crucial mechanisms especially from GD to GO remain unclear. At present, the study of GO epigenetics focuses primarily on only one kind of epigenetic modifications, but the role of its regulatory network in controlling molecular mechanisms of GO remains unknown. Therefore, there is an unmet need to conduct a combined analysis of the varying epigenetic modifications that can further explore the possible roles of various less explored epigenetic factors that have been implicated in the pathogenesis of GO.

Epigenetic inheritance can link environmental factors and diseases. How environmental factors can lead to the progression of GO by facilitating epigenetic inheritance and intestinal microbial dysregulation in GO remains to be elucidated. To date, GO is clinically diagnosed by an ophthalmologist with an adjunct imaging such as CT or magnetic resonance imaging (MRI). A few studies have shown that some epigenetic markers and intestinal microflora might be different in GD patients with or without GO. However, it is still difficult to predict which GD patients will develop into GO or severe GO, and which GD patients will be responsive or unresponsive to the different types of treatment. Further studies should focus on the current challenges in the future.

## Author Contributions

YW and H-SY participated in the conceptualization and writing. X-MM, XW, XS, L-JW, X-QL, and X-YL participated in the review and editing. All authors contributed to the article and approved the submitted version.

## Funding

This work was supported by National Key R&D Program of China (grant number: 2018YFC1004300), National Natural Science Foundation Project of China (grant number: 82160154, 81670844), the Key Project of Guizhou Provincial Science and Technology Department (grant number: QKH-JC-2019-1464), the Excellent Talent Support Program of Guizhou Provincial Education Department (grant number: QJH-KY-2017-077), the Science and Technology Foundation of Guizhou Province (grant number: QKH-PTRC-2018-5772-042), the Science and Technology Project of Zunyi (grant number: ZSKH-HZ-2020-35), and the Program for Excellent Young Talents of Zunyi Medical University (grant number: 18-ZY-001).

## Conflict of Interest

The authors declare that the research was conducted in the absence of any commercial or financial relationships that could be construed as a potential conflict of interest.

## Publisher’s Note

All claims expressed in this article are solely those of the authors and do not necessarily represent those of their affiliated organizations, or those of the publisher, the editors and the reviewers. Any product that may be evaluated in this article, or claim that may be made by its manufacturer, is not guaranteed or endorsed by the publisher.
